# Assessing the relationship between delay discounting and decisions to engage in various protective behaviors during COVID-19

**DOI:** 10.1186/s41235-024-00566-6

**Published:** 2024-06-18

**Authors:** Julia G. Halilova, Samuel Fynes-Clinton, Donna Rose Addis, R. Shayna Rosenbaum

**Affiliations:** 1 York University, 4700 Keele St., Toronto, ON M3J 1P3 Canada; 2 Rotman Research Institute, Baycrest Hospital, North York, Canada; 3 University of Toronto, Toronto, Canada; 4 The University of Auckland, Auckland, New Zealand

**Keywords:** Decision making, Delay discounting, Public health measures, Vaccination, COVID-19

## Abstract

Research suggests that discounting of delayed rewards (i.e., tendency to choose smaller immediate rewards over large later rewards) is a promising target of intervention to encourage compliance with public health measures (PHM), such as vaccination compliance. The effects of delay discounting, however, may differ across the types of PHMs, given that the benefits of vaccination, unlike other PHMs (physical distancing, handwashing, and mask-wearing), are more temporally delayed. Here, we examined whether delay discounting predicts engaging in COVID-19 PHMs in approximately 7,000 participants recruited from 13 countries in June–August 2021. After controlling for demographic and distress variables, delay discounting was a negative predictor of vaccination, but a positive predictor of physical distancing (when restrictions are in place) and handwashing. There was no significant association between delay discounting and frequency of mask-wearing. It is possible that increasing vaccination compliance may require greater emphasis on future benefits of vaccination, whereas promotion of physical distancing and hand hygiene may require greater focus on the present moment. Further research is needed to investigate the nature of this relationship and its implications for public health messaging.

**Supplementary Information:**

The online version contains supplementary material available at 10.1186/s41235-024-00566-6.

## Introduction

Widespread compliance with public health measures (PHMs) has been critical to containing the COVID-19 pandemic as well as other infectious diseases. Vaccination, mask-wearing, handwashing, and physical distancing are among the most commonly recommended PHMs designed to mitigate the impacts of the pandemic (World Health Organization, 2020). Widespread public compliance with PHMs is necessary to prevent severe illness and death, and alleviate hospital burden during pandemics, and is of increasing importance in light of global population aging. Understanding decisions to engage in protective behaviors during COVID-19 will help inform ways to encourage PHM compliance, with important implications for managing future pandemics.

Here, we investigate a well-established behavioral measure of decision making as a predictor of compliance with different PHMs during the COVID-19 pandemic. Delay discounting is a phenomenon where the subjective value of rewards decreases as a function of delay, such that individuals discount the worth of future rewards relative to immediate ones (Green & Myerson, 2004). Steep or high delay discounting indicates a tendency for immediate gratification and is often associated with impulsivity (Moreira & Barbosa, 2019). This tendency is associated with financial instability (Ruggeri et al., 2022) and problematic health behaviors (e.g., Barlow et al., 2016; Bickel et al., 2019; Daugherty & Brase, 2010; Robles et al., 2011; Rung & Madden, 2018), including those that were heightened during the COVID-19 pandemic (Cutler & Summers, 2020; Halilova et al., 2022; Wu et al., 2021; Zhang & Chen, 2021). By contrast, individuals with shallow or low delay discounting tend to exhibit greater levels of self-control and be better equipped to make decisions that align with their longer-term goals (e.g., Bickel et al., 2018).

There are several reasons why delay discounting is a promising behavioral economic measure for public health purposes and one that may be leveraged to address health crises on a global scale. Incorporating behavioral economic measures, such as delay discounting, into forecasting models of disease spread could enhance the accuracy of predictions and allow for the implementation of targeted interventions to mitigate the spread of COVID-19 (Marin-Lopez et al., 2022). Furthermore, unlike self-report measures that explicitly ask participants to reflect on their decision-making tendencies, delay discounting tasks measure decision-making behavior in a less conspicuous and more objective way (Myerson et al., 2001). As such, these tasks do not rely on participants’ awareness of their decision-making behaviors and are less likely to be affected by social desirability and other response biases (Ruggeri et al., 2024).

The promise of delay discounting as a predictor of compliance with COVID-19 PHMs has been recognized in recent research efforts involving diverse populations from different countries, tested at different times throughout the pandemic (Agrawal et al., 2023; Byrne et al., 2021; Calluso et al., 2021; DeAngelis et al., 2022; Halilova et al., 2022; Hudson et al., 2022; Krawiec et al., 2022; Lloyd et al., 2021; Strickland et al., 2022; Wismans et al., 2021). There is consistency in findings of a negative relationship between delay discounting and vaccination attitudes and status (Halilova et al., 2022; Hudson et al., 2022; Strickland et al., 2022). The relationship between delay discounting and other PHMs (e.g., handwashing/cleaning, physical distancing, and mask-wearing), however, has yielded mixed findings, with some studies showing a negative relationship between discounting of delayed rewards and PHMs (Agrawal et al., 2023; Byrne et al., 2021; DeAngelis et al., 2022; Lloyd et al., 2021), other studies showing a positive relationship between delay discounting and PHM compliance (Calluso et al., 2021; Wismans et al., 2021), and yet other studies indicating no relationship between delay discounting and compliance (Krawiec et al., 2022). As shown in Table 1, the picture is complicated by the study-specific differences, including being conducted in different populations at different time points during the pandemic under varying conditions that were evolving quickly. Moreover, the type of PHM examined and the delay discounting measure used varied across studies. In the current study, we investigated the relationships between delay discounting and multiple PHMs in the same sample of participants, and we recruited sufficient N’s per country (at least ~ 100 per country) to assess whether the effect generalized across countries. We measured delay discounting using the AuC, which is considered to be an atheoretical measure, unlike log-adjusted k values that other studies in this literature have used (Calluso et al., 2021; DeAngelis et al., 2022; Krawiec et al., 2022; Wismans et al., 2021).

**Table 1:** Studies examining the relationship between delay discounting and PHM compliance during the COVID-19 pandemic

Study	PHM(s) studied	Measure of discounting	*n*	Countr(ies) of data collection	Time of data collection	Correlation of delay discounting with PHMs
Byrne et al., 2021	Physical distancing, mask-wearing	Area-under-the-curve	404	USA	July–December 2020	Greater delay discounting associated with less physical distancing and mask-wearing
Lloyd et al., 2021	Physical distancing	Delay discounting magnitude effect slope (*m*), and its intercept (*c*)	442	UK	April–May, 2020	Greater delay discounting predicted poorer adherence to physical distancing measures
DeAngelis et al., 2022	Physical distancing, stockpiling	Log-transformed *k* value	3,686	96 countries	March–May 2020	Discounting negatively correlated with physical distancing and positively correlated with stockpiling
Calluso et al., 2021	Going out, hand sanitation, use of protective equipment	Log-transformed *k* value	353	Italy	May 2020	Discounting rate was positively related to compliance physical distancing and mask- and glove-wearing
Wismans et al., 2021	Social distancing, hygiene	Log-transformed *k* value	6,759	Belgium, France, Ireland, Italy, the Netherlands, Portugal, Sweden	June 2020	Discounting rate positively related to social distancing and hygiene compliance
Krawiec et al., 2022	Physical distancing, mask-wearing, disinfection	Log-transformed *k* value	338	Poland	December 2020–February 2021	No significant correlation between delay discounting and any of the PHMs studied
Agrawal et al., 2023	Social distancing, mask-wearing	Area-under-the-curve	12,906	USA	March 26, 2020; April 2020; June 30, 2020; November 2, 2020	Discounting rate negatively related to compliance with physical distancing, but not mask-wearing

Delay discounting emerges across studies as a negative predictor of vaccination status, but the relationship between discounting and other PHMs is less consistent. In addition to the study-specific factors described above, it is possible that there is a more fundamental reason for the different relationships between delay discounting and vaccination versus other PHMs. It is possible that when deciding whether to engage in PHMs, an individual may consider shorter- and longer-term benefits of engaging in those behaviors (e.g., immediate side effects of a vaccine vs. long-term immunity). Importantly, the effect of delay discounting may differ across types of protective behaviors depending on the perceived temporal delay of PHM benefits, which may help to explain the mixed findings in the literature. For example, it has been well publicized that it takes approximately 2 weeks to develop immunity to the virus after receiving the vaccine and the benefits last for months, whereas physically distancing from others results in immediate reduction of virus transmission that is limited to that specific time (e.g., Bernal et al., 2021; Chea et al., 2021; Sun et al., 2022).

The current research examined the relationship between delay discounting and compliance in different PHMs (i.e., vaccination status, handwashing/cleaning, physical distancing, and mask-wearing) in mid-2021 in a large sample of adults from 13 countries that continued to promote PHMs in the face of the ongoing COVID-19 pandemic. To our knowledge, this is the first study to investigate the relationships between delay discounting and vaccination vs. other PHMs in the same sample of participants. We predicted that, after controlling for demographic variables, psychological distress, and intolerance of uncertainty, greater discounting of delayed rewards (i.e., more short-sighted thinking) would be associated with a reduced likelihood of being vaccinated but increased frequency of engaging in other PHMs that provide more immediate benefits, such as handwashing and cleaning, physical distancing, and mask-wearing. The multinational sample also allowed us to explore the consistency of the relationship between the variables across 13 industrialized countries that varied in pandemic severity, vaccination rates, and implementation of PHMs.

## Method

### Participants

Recruitment was conducted through an online platform (Prolific.co) from June 27, 2021 to August 31, 2021.^1^ Using Prolific’s built-in inclusion/exclusion filters, the study was available only to users meeting the following inclusion criteria: aged 18 years or older, fluent in English, normally residing in one of 14 target countries^2^ across North America, Europe, Australasia, and Africa, and free from neurological impairments or learning disabilities. Target countries were selected with the goal of capturing varying COVID-19 impact severity and a range of government mandates in place at the time of testing (Mathieu et al., 2020). Countries with fewer than 200 active participants on the recruitment platform were not included in the target list. Of the 7,667 participants who provided informed consent, data from 320 individuals were excluded due to failure to meet inclusion criteria (e.g., currently residing in a non-targeted country); non-completion of the survey (i.e., those who completed less than 95% of the survey); and/or responding incorrectly to more than one attention check item (see below). Data from 421 participants from South Africa were excluded due to a substantially different approach in government response, limited vaccine availability, and additional obstacles to compliance with PHMs (e.g., lack of access to clean water; Staunton et al., 2020). The final data set was composed of 6,926 participants who were on average 28.62 (*sd* = 10.18) years old; 58% were female, 40% male, and 2% non-binary. Approximately 35% of the sample had achieved secondary level education, 48% had an undergraduate degree, and 16% of the sample achieved postgraduate education. Approximately 23% of the sample self-identified as essential workers. Average subjective rating of relative income among participants (on a 100-point sliding scale; Adler et al., 2000; Smith et al., 2019) was 36.31 (*sd* = 23.8).

### Measures

#### PHMs during COVID-19

Participants were asked a series of questions about their compliance with protective behaviors during COVID-19. Participants chose between five options in response to the question about their vaccination status: 1 = yes, I have received all necessary doses, 2 = yes, although I require another dose, 3 = no, but I am planning to get vaccinated, 4 = no, I am not planning to get vaccinated, 5 = prefer not to say. A binary *vaccination status* variable was created, distinguishing between those who were vaccinated (fully or partially) or not (including both those who were planning and not planning to get vaccinated in the future). Participants were asked to indicate on a 5-point scale the frequency with which they engaged in following five behaviors over the past week: (1) physical contact with (i.e., touching) and (2) being in close proximity with people they do not live with (0 = never, 1 = once, 2 = every several days, 3 = daily, 4 = more than once daily); (3) cleaning and disinfecting frequently touched surfaces (e.g., tables, doorknobs, light switches; 0 = not at all, 1 = every several days, 2 = every other day, 3 = daily, 4 = more than once daily); (4) cleaning their hands with sanitizer or soap and water (0 = never, 1 = a few times a week, 2 = daily, 3 = several times a day, 4 = at least once an hour); and (5) mask-wearing (0 = never, 1 = once, 2 = every several days, 3 = daily, 4 = more than once daily). The mask-wearing item and two composite variables—*physical distancing* (sum of reverse-coded physical contact and close proximity items), and *cleaning* (sum of cleaning and disinfecting frequently touched surfaces and cleaning hands)—were used in analyses.

#### Delay discounting task

In this well-established intertemporal choice procedure (Ciaramelli et al., 2021; Halilova et al., 2022; Mok et al., 2020), participants viewed pairs of monetary amounts and were asked to choose between smaller, immediate rewards which varied between trials, and a larger, delayed reward of $2,000. Participants were asked to make six choices at each of seven delays for the larger reward (waiting 1 week, 1 month, 3 months, 6 months, 1 year, 3 years, and 10 years before receiving the $2000 reward). An iterative, adjusting-amount procedure was used in which the amount of the immediate reward was increased or decreased based on the participant’s previous choice at that delay, converging on the amount of the immediate reward equivalent in subjective value to the delayed reward. The first adjustment was half of the difference between the immediate and delayed amounts presented on the first trial, with each subsequent adjustment being half of the preceding adjustment. For example, in the condition where a future reward of $2000 could be received in 3 years, the first choice presented to the participants would be “$1000 right now or $2000 in 3 years.” If the participant chose “$2000 in 3 years,” the choice on the second trial would be “$1500 right now or $2000 in 3 years.” If the participant then chose “$1500 right now”, the choice on the third trial would be “$1250 right now or $2000 in 3 years.” Following the sixth and final trial of each condition, the subjective value of the delayed reward was estimated as the amount of the immediate reward that would be presented on a seventh trial. A larger subjective value of the delayed reward indicated less discounting of delayed rewards. A smaller subjective value indicated a more short-sighted decision making. Degree of discounting was measured by examining the subjective values of reward across the seven delays and computing *Area-under-the-curve* (AuC), a single, theoretically-neutral measure of discounting (Myerson et al., 2001). Another advantage of using AuC as the measure of delay discounting is that it tends to generate approximately normally distributed scores (Myerson et al., 2001). The scores range from 0 to 1, with lower AuC representing a greater discounting rate (i.e., more short-sighted decision making).

#### Demographic questionnaire

Participants completed a demographic questionnaire assessing current country of residence, age, gender (female/male/non-binary), highest level of education obtained (no schooling/primary schooling/secondary schooling/undergraduate degree or professional equivalent/postgraduate degree), essential worker status (yes/no), and personal income. For essential worker status, participants indicated if they worked in an occupations supplying critical services during the pandemic: government; health and safety (e.g., healthcare, emergency response); utilities (e.g., water, energy, sanitation, transport, communications); food (e.g., supermarkets); and manufacturing. A subjective measure of relative income was used, such that participants estimated their current income on a sliding scale (0–100) marked by points representing low (0), average (50), and high (100) incomes in their own country/region (Adler et al., 2000; Smith et al., 2019).

#### Psychological distress index

Presence and severity of anxiety and depressive symptoms were assessed with the Generalized Anxiety Disorder 7-item (GAD-7) scale (Spitzer et al., 2006) and the Patient Health Questionnaire 9-item (PHQ-9) scale (Kroenke et al., 2001), respectively. Participants rated the frequency of symptoms experienced over the past two weeks on a four-point scale (0 = not at all; 3 = nearly every day). For each scale, a total score was computed, where higher scores reflect more severe symptoms. Total scores from these measures were standardized and then summed to create a *psychological distress index*.

#### Intolerance of uncertainty scale (IUS-12)

The IUS-12 is a 12-item measure of one’s difficulties tolerating uncertainty (Carleton et al., 2007). Participants used a 6-point scale (0 = not at all characteristic of me; 5 = entirely characteristic of me) to respond to items measuring two factors of intolerance of uncertainty: prospective anxiety, the cognitive component of intolerance of uncertainty that indicates one’s tendency to worry about future events (e.g., “I always want to know what the future has in store for me”) and inhibitory anxiety, the behavioral component of intolerance of uncertainty that represents avoidance tendencies in the face of uncertainty (e.g., “I must get away from all uncertain situations”; Carleton et al., 2007). *Intolerance of uncertainty* score was calculated as a sum of participants’ responses to IUS-12, ranging from 0 to 60.

#### Attention checks

Three items from the Conscientious Responder Scale (CRS; Marjanovic et al., 2014) were included at select points within the survey to identify random responders (e.g., “To answer this question, please choose option three, neither agree nor disagree.”).

## Results

**Fig. 1:**
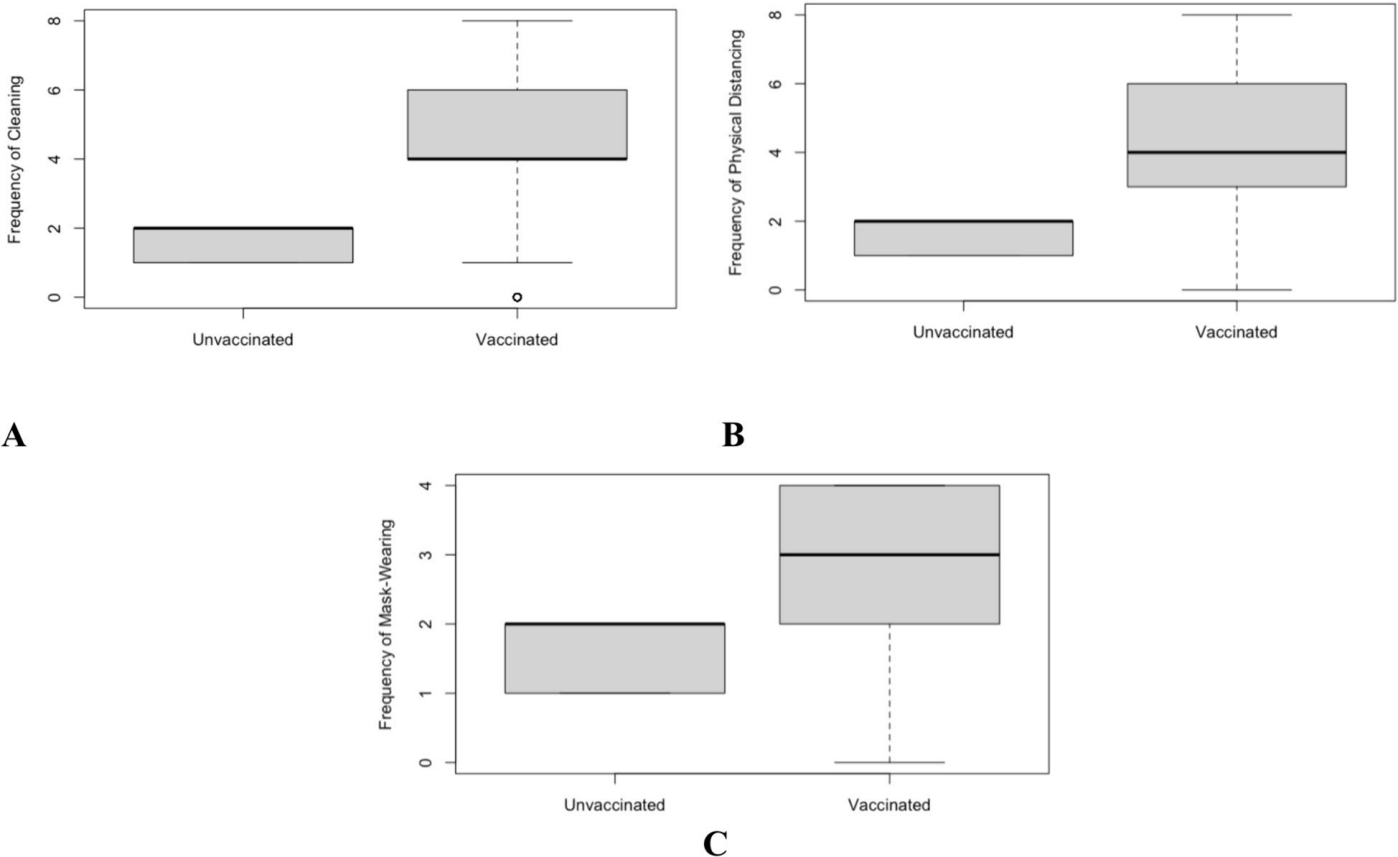
**A** Frequency of engaging in physical distancing behaviors by vaccination status. **B** Frequency of mask-wearing by vaccination status. **C** Frequency of cleaning behaviors by vaccination status

Descriptive statistics of the variables, including the frequency of engaging in physical distancing, cleaning, and mask-wearing broken down by vaccination status, are provided in Table 2 and Fig. 1. Country-level summary data are provided in Supplementary Materials. The correlations between physical distancing, cleaning, and mask-wearing are provided in Table 3.

**Table 2:** Descriptive data summary

Variables	Vaccinated (n = 4355)	Unvaccinated (n = 2571)
Age	29.58 (10.95)	27.01 (8.49)
Gender (female/male/non-binary)	2661/1597/93	1333/1173/41
Education level (no education/primary/secondary/undergraduate/postgraduate)	5/12/1495/2090/752	4/5/936/1249/357
Income (0–100)	37.74 (24.15)	35.21 (23.80)
Essential workers *(% yes)*	26.96%	16.06%
Intolerance of uncertainty	35.19 (9.28)	35.04 (9.31)
Area-under-curve	0.41 (0.25)	0.37(0.25)
Psychological distress	− 0.03 (1.88)	0.04 (1.89)
Cleaning *(Range; Median)*	0–8; 4	0–8; 4
Physical distancing *(Range; Median)*	0–8; 4	0–8; 5
Mask-wearing *(Median)*	0–4; 3	0–4; 3

**Table 3:** Correlations among PHMs

	Cleaning	Mask-wearing
Physical distancing	− 0.01	− 0.01
Cleaning	–	0.22

### Vaccination

**Fig. 2:**
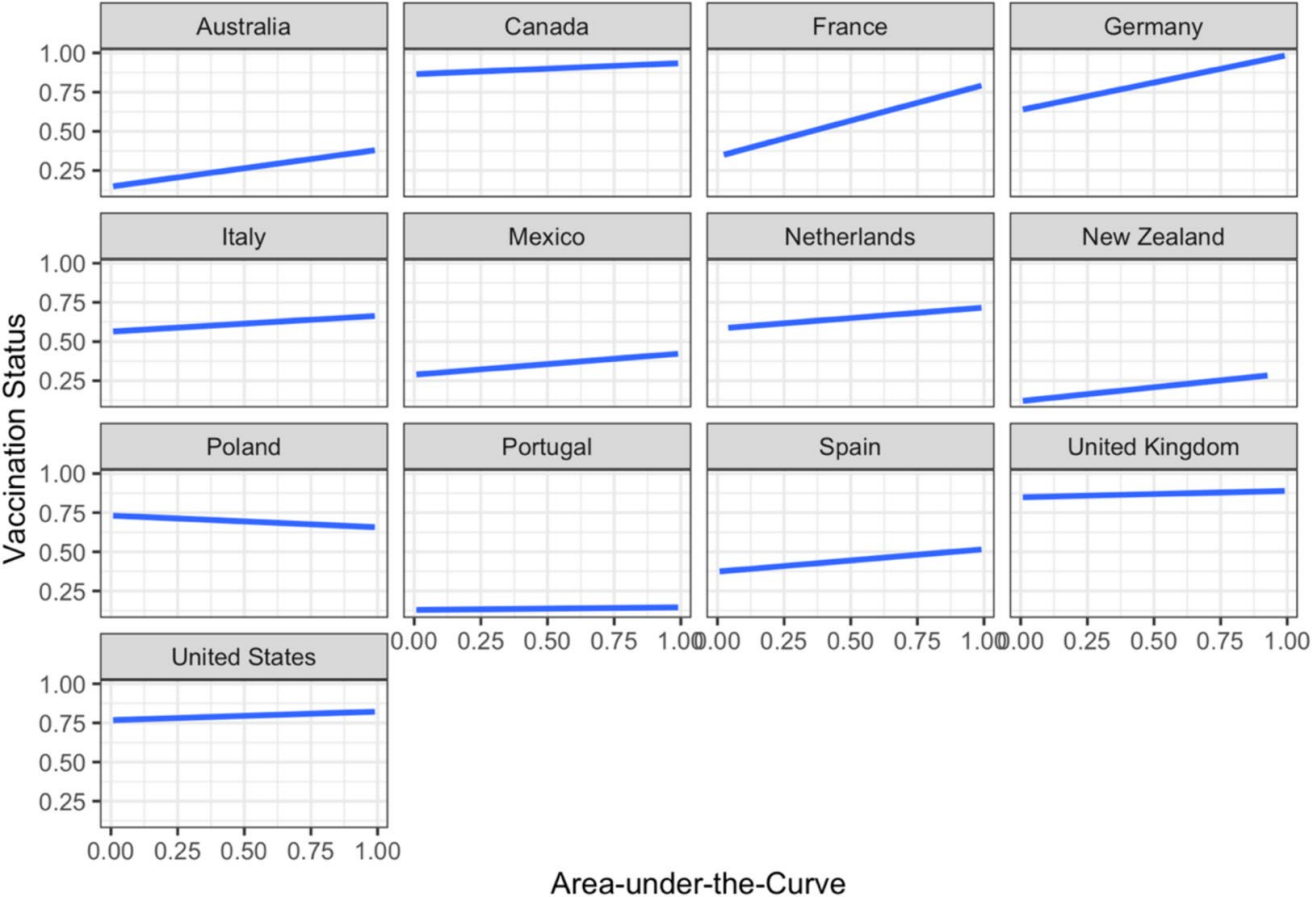
Vaccination status (0 = unvaccinated, 1 = vaccinated) plotted by area-under-the-curve across the 13 countries from where the data were collected. The plots indicate generally positive relationships between the area-under-the-curve and vaccination status across countries, except for Poland where the relationship was negative

A multilevel logistic regression model was constructed using R packages *lme4* (Bates et al., 2012) and *lmerTest* (Kuznetsova et al., 2017) with vaccination status (unvaccinated vs. vaccinated) as the outcome variable, and age, gender, education level, income, psychological distress index, essential worker status, intolerance of uncertainty, and AuC as predictors. To account for possible systematic differences across countries (e.g., COVID-related severity, population vaccination rates, government response), each participant’s vaccination status (Level 1) was nested within country (Level 2). A likelihood ratio test showed that the model accounted for significantly more variance in the data compared to an unconditional intercept-only model, *χ*^2^*(13)* = 225.77, *p* < 0.001. The tendency to choose larger future rewards over smaller immediate ones (larger AuC) significantly increased the odds of being vaccinated after controlling for other variables in the model (*p* < 0.001; Table 4). All of the continuous control variables in the model positively predicted the likelihood of being vaccinated (*p* < 0.05), with the exception of the psychological distress index (*p* = 0.754) and intolerance of uncertainty (*p* = 0.204), which were not significant predictors. After controlling for other variables, the results show that self-identifying as a male, compared to female, was associated with a significantly lower likelihood of being vaccinated. After controlling for other variables in the model, completing postgraduate studies compared to undergraduate studies was associated with greater likelihood of being vaccinated. The association between AuC and vaccination status was further explored by country. As evident in Fig. 2, the association between AuC and vaccination status was stronger in countries where the participant vaccination rate was moderate (e.g., France, Germany, Italy, the Netherlands, Australia). In contrast, this association appears weaker in countries where the participant vaccination rate was either very high (e.g., Canada, USA, and UK) or very low (e.g., Portugal) in our sample represented by nearly horizontal lines at the top or bottom of the graphs in Fig. 1, respectively. Poland appears to be the only country in our sample that showed a negative relationship between AuC and vaccination status.

**Table 4:** Results of the multilevel logistic regression model predicting vaccination status

Fixed effects	*b*	*SE*	*z*	*p*	OR	95% CI
Intercept	0.26	0.35	0.72	.469	1.29	[0.65, 2.58]
Age^†^	0.28	0.04	7.74	< .001	1.32	[1.23, 1.42]
Gender (male)	− 0.31	0.06	− 4.83	< .001	0.73	[0.65, 0.83]
Gender (non-binary)	0.32	0.22	1.44	.149	1.38	[0.89, 2.14]
Education level (no schooling)	− 0.80	0.76	− 1.06	.287	0.45	[0.10, 1.97]
Education level (primary)	0.24	0.60	0.40	.691	1.27	[0.39, 4.13]
Education level (secondary)	− 0.05	0.07	− 0.74	.459	0.95	[0.83, 1.09]
Education level (postgraduate)	0.33	0.09	3.60	< .001	1.39	[1.16, 1.66]
Relative income^†^	0.08	0.03	2.41	.016	1.08	[1.02, 1.15]
Essential worker status	0.51	0.08	6.43	< .001	1.67	[1.43, 1.95]
Psychological distress^†^	− 0.01	0.04	− 0.31	.754	0.99	[0.92, 1.06]
Intolerance of uncertainty^†^	0.04	0.04	1.27	.204	1.05	[0.98, 1.12]
Delay discounting (AuC)^†^	0.14	0.03	4.52	< .001	1.15	[1.08, 1.22]

Random effects	Estimate	***SD***
Intercept error variance (country)	1.58	1.26

*Note*. ^†^ The variable was standardized. AuC = Area-under-the-curve, CI = confidence interval; OR = odds ratio; SD = standard deviation; SE = standard error of the mean. Female was used as the reference category for gender. Undergraduate level of education was used as the reference category for level of education

### Cleaning

**Fig. 3:**
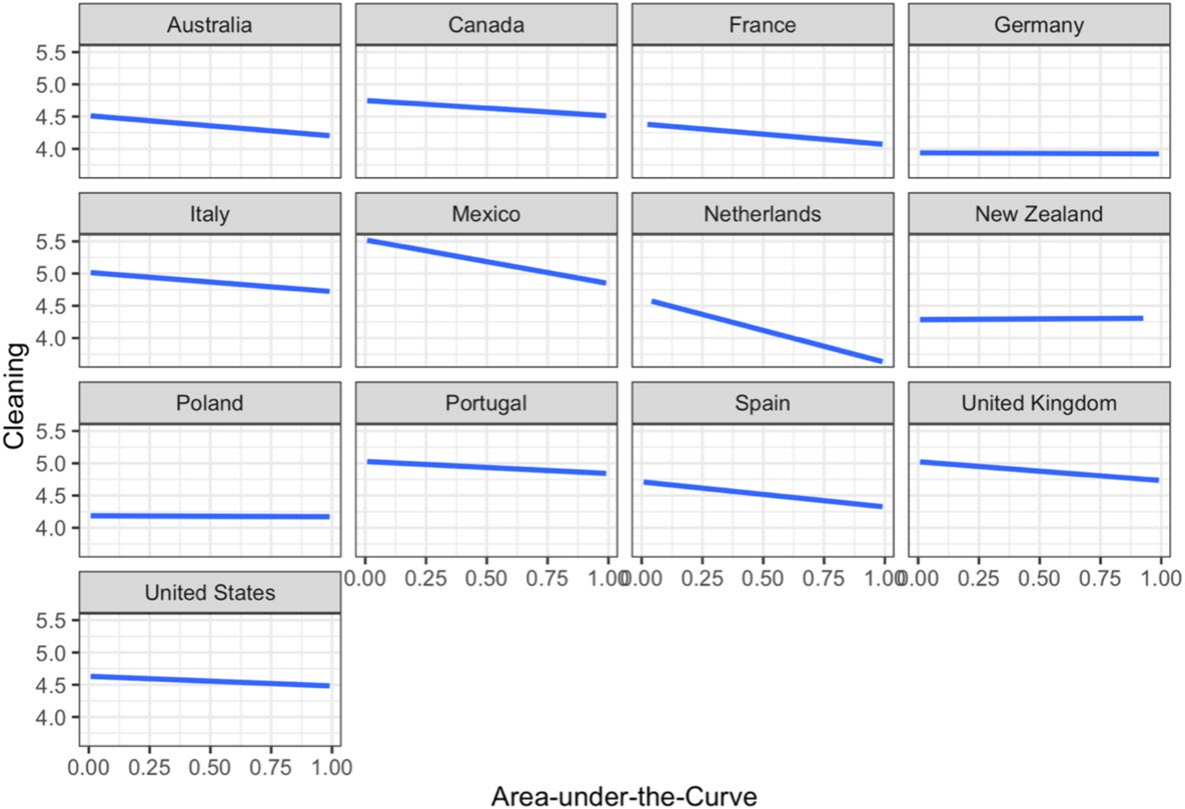
Frequency of cleaning behaviors, ranging from 0 to 8, plotted by area-under-the-curve across the 13 countries from where the data were collected. The plots indicate generally negative relationships between the area-under-the-curve and frequency of cleaning behaviors across countries, except for Germany, Poland, and New Zealand where there is no association between these variables

Given that the remaining PHMs are measured on an ordinal scale, *ordinal* R package (Christensen, 2023) was used to implement cumulative link mixed model analysis. A multilevel model was constructed with the cleaning composite score as the outcome variable, and age, gender, education level, income, psychological distress index, essential worker status, intolerance of uncertainty, vaccination status, and AuC as predictors. Each participant’s frequency of cleaning behaviors (Level 1) nested within country (Level 2). A likelihood ratio test showed that the model accounted for significantly more variance in the data compared to an unconditional intercept-only model, *χ*^2^(*14*) = 160.72, *p* < 0.001. AuC negatively predicted the frequency of cleaning and handwashing after controlling for other variables in the model (*p* < 0.001; Table 5), indicating that shorter-sighted thinking was associated with increased engagement in cleaning behaviors. In contrast, female gender (compared to male), relative income, essential worker status, psychological distress, intolerance of uncertainty, and vaccination status were associated with a higher frequency of cleaning behaviors. The association between AuC and frequency of engaging in cleaning was further explored by country. As evident in Fig. 3, the relationship between the variables was negative in 10 out of 13 countries, while there was no relationship between AuC and frequency of engaging in cleaning behaviors in Germany, New Zealand, and Poland.

**Table 5:** Results of the cumulative link mixed model predicting frequency of cleaning behaviors

Coefficients	*b*	*SE*		*z*	*P*	OR	95% CI
Age^†^	0.03	0.02		1.38	.168	1.03	[− 0.01, 0.08]
Gender (male)	− 0.31	0.05		− 6.71	< .001	0.73	[− 0.41, − 0.22]
Gender (non-binary)	− 0.13	0.15		− 0.83	.408	0.88	[− 0.43, 0.17]
Education level (no schooling)	− 2.48	0.61		− 4.04	< .001	0.08	[− 3.68, − 1.28]
Education level (primary)	0.31	0.46		0.68	.499	1.36	[− 0.59, 1.21]
Education level (secondary)	− 0.05	0.05		− 0.94	.347	0.95	[− 0.14, 0.05]
Education level (postgraduate)	− 0.10	0.06		− 1.54	.125	0.91	[− 0.22, 0.03]
Relative income^†^	0.07	0.02		2.96	.003	1.07	[0.02, 0.12]
Essential worker status	0.18	0.05		3.49	< .001	1.20	[0.08, 0.29]
Psychological distress^†^	0.09	0.03		3.51	< .001	1.10	[0.04, 0.14]
Intolerance of uncertainty^†^	0.07	0.03		2.77	.005	1.07	[0.02, 0.12]
Vaccination Status	0.16	0.05		3.06	.002	1.18	[0.06, 0.27]
Delay discounting (AuC)^†^	− 0.08	0.02		− 3.75	< .001	0.92	[− 0.13, − 0.04]

Random effects	Estimate		***SD***
Intercept error variance (country)	0.21	0.46	

^†^ The variable was scaled to improve model fit. AuC = Area-under-the-curve; CI = confidence interval; SD = standard deviation; SE = standard error of the mean. Female was used as the reference category for gender. Undergraduate level of education was used as the reference category for level of education. Unvaccinated was used as the reference category for vaccination status

### Physical distancing

**Fig. 4:**
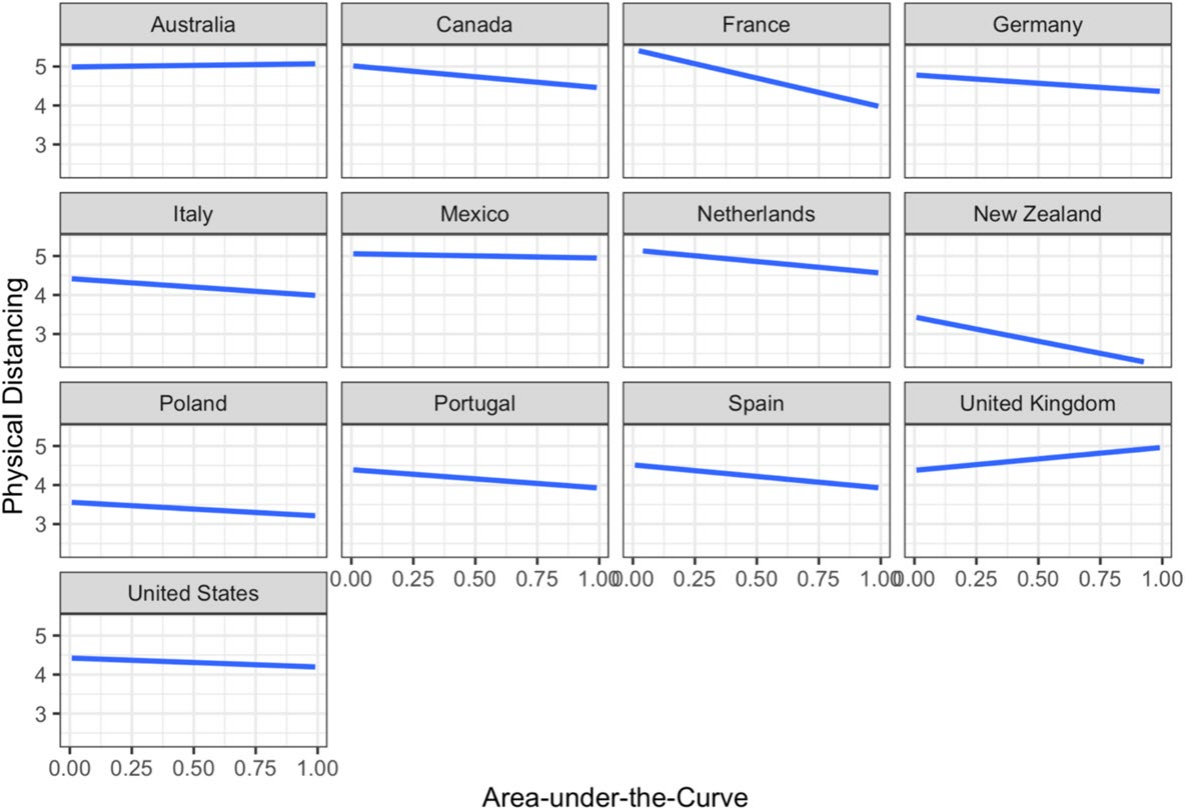
Frequency of physical distancing behaviors, ranging from 0 to 8, plotted by area-under-the-curve across the 13 countries from where the data were collected. The plots indicate generally negative relationships between the area-under-the-curve and frequency of cleaning behaviors across countries, except for UK, where the relationship between the variables is positive

A cumulative link mixed model was constructed with the physical distancing composite score as the outcome variable, and the same set of predictors and the same structure of random effects as in the earlier models. A likelihood ratio test shows that the model accounted for significantly more variance in the data compared to an unconditional intercept-only model, *χ*^2^(*14*) = 477.57, *p* < 0.001. Delay discounting (AuC) did not significantly predict the frequency of physical distancing after controlling for other variables in the model (*p* = 0.106; Table 6). Age, income, essential worker status, psychological distress, and intolerance of uncertainty significantly predicted the frequency of engaging in physical distancing (Table 6). The association between AuC and frequency of physical distancing behaviors was explored by country (Fig. 4). The UK was the only country with a positive slope in the relationship between AuC and physical distancing; notably this was also the only country in the sample that did not have physical distancing restrictions in place during the time of data collection (Mathieu et al., 2020). Running the model again without the UK data resulted in AuC becoming a significant negative predictor of frequency of physical distancing, *b* = − 0.06, *SE* = 0.02, *z* = − 2.35, *p* = 0.019, OR = 0.95, 95% CI [− 0.10, − 0.01], indicating that shorter-sighted thinking was associated with increased engagement in physical distancing. It was also notable that the associations between physical distancing and AuC were strongest in France, Spain, and Portugal (Fig. 4), countries with the most stringent physical distancing mandates (i.e., stay-at-home was a requirement with the exception of essentials) in place during the time of data collection (Mathieu et al., 2020).

**Table 6:** Results of the cumulative link mixed model predicting frequency of physical distancing behaviors

Coefficients	*b*	*SE*	*z*	*p*	OR	95% CI
Age^†^	0.27	0.02	11.38	< .001	1.31	[0.22, 0.32]
Gender (male)	− 0.04	0.05	− 0.95	.344	0.96	[− 0.13, 0.05]
Gender (non-binary)	0.36	0.15	2.35	.019	1.43	[0.06, 0.65]
Education level (no schooling)	− 0.31	0.53	− 0.58	.565	0.74	[− 1.35, 0.74]
Education level (primary)	0.92	0.44	2.10	.036	2.51	[0.06, 1.78]
Education level (secondary)	− 0.05	0.05	− 1.03	.301	0.95	[− 0.15, 0.05]
Education level (postgraduate)	0.14	0.06	2.17	.030	1.15	[0.01, 0.26]
Relative income^†^	− 0.11	0.02	− 4.76	< .001	0.90	[− 0.15, − 0.06]
Essential worker status	− 0.83	0.05	− 15.68	< .001	0.43	[− 0.94, − 0.73]
Psychological distress^†^	0.05	0.03	1.87	.062	1.05	[− 0.01, 0.10]
Intolerance of uncertainty^†^	0.14	0.02	5.46	< .001	1.15	[0.09, 0.19]
Vaccination Status	− 0.10	0.05	− 1.83	.068	0.91	[− 0.20, 0.01]
Delay discounting (AuC)^†^	− 0.04	0.02	− 1.62	.106	0.97	[− 0.08, 0.01]

Random effects	Estimate	*SD*
Intercept error variance (country)	0.26	0.51

^†^The variable was standardized. AuC = Area-under-the-curve; CI = confidence interval; SD = standard deviation; SE = standard error of the mean. Female was used as the reference category for gender. Undergraduate level of education was used as the reference category for level of education. Unvaccinated was used as the reference category for vaccination status

### Mask-wearing

**Fig. 5:**
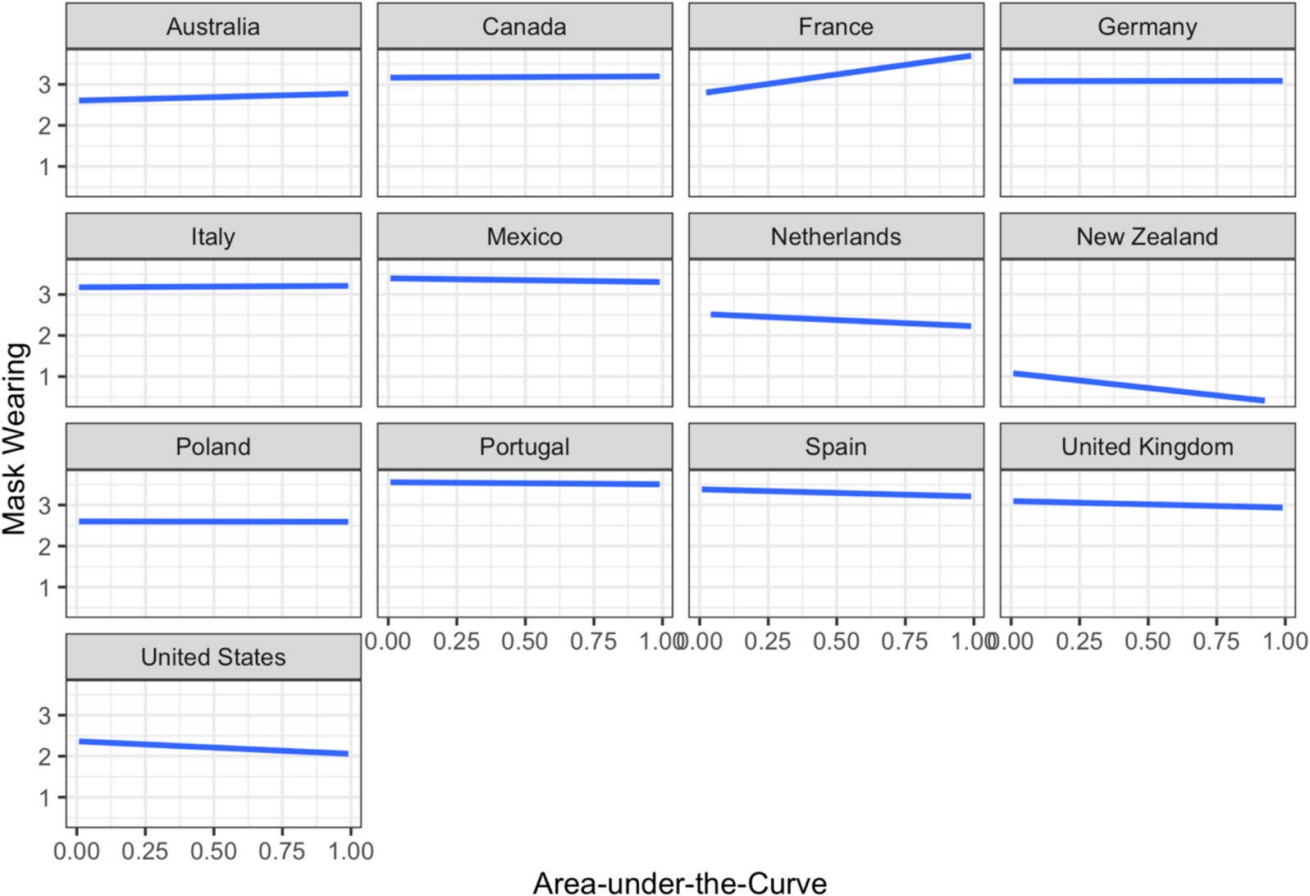
Frequency of mask-wearing behavior, ranging from 0 to 4, plotted by area-under-the-curve across the 13 countries from where the data were collected. The plots indicate generally no significant relationships between the area-under-the-curve and frequency of mask-wearing behavior across countries

A multilevel model was constructed with mask-wearing as the outcome variable and the same set of predictors and the same structure of random effects as the earlier models. A likelihood ratio test showed that the model accounted for significantly more variance in the data compared to an unconditional intercept-only model, *χ*^2^(*14*) = 202.77, *p* < 0.001. Delay discounting (AuC) did not significantly predict the frequency of mask-wearing after controlling for other variables in the model (*p* = 0.071; Table 7). Among control variables, gender (female compared to male), essential worker status, psychological distress, and vaccination status were significant positive predictors of frequency of mask-wearing. On the other hand, age was a significant negative predictor of mask-wearing in the model. The association between AuC and frequency of mask-wearing was explored by country (Fig. 5). There were no strong relationships between delay discounting and mask-wearing among participants from most countries in our sample. In our sample, France and New Zealand appear as the countries with the strongest associations between AuC and mask-wearing, positive and negative, respectively. However, the association is not significant when fitting the model with the same set of control variables individually for these countries (for France, *b* = 0.36, *p* = 0.10 and for New Zealand, *b* = − 0.22, *p* = 0.15).

**Table 7:** Results of the cumulative link mixed model predicting frequency of mask-wearing behavior

Coefficients	*b*	*SE*	*z*	*p*	OR	95% CI
Age^†^	− 0.07	0.03	− 2.79	.005	0.93	[− 0.12, − 0.02]
Gender (male)	− 0.16	0.05	− 3.37	< .001	0.85	[− 0.30, − 0.07]
Gender (non-binary)	0.24	0.17	1.43	.153	1.27	[− 0.09, 0.57]
Education level (no schooling)	− 1.06	0.56	− 1.87	.059	0.35	[− 2.15, 0.04]
Education level (primary)	− 0.27	0.45	− 0.61	.539	0.76	[− 1.15, 0.60]
Education level (secondary)	− 0.09	0.05	− 1.81	.071	0.91	[− 0.20, 0.01]
Education level (postgraduate)	0.05	0.07	0.79	.429	1.05	[− 0.08, 0.18]
Relative income^†^	0.01	0.02	0.32	.753	1.01	[− 0.04, 0.06]
Essential worker status	0.37	0.06	6.63	< .001	1.45	[0.26, 0.48]
Psychological distress^†^	0.13	0.03	4.81	< .001	1.14	[0.08, 0.19]
Intolerance of uncertainty^†^	0.02	0.02	0.75	.450	1.02	[− 0.03, 0.07]
Vaccination status	0.44	0.06	7.72	< .001	1.55	[0.33, 0.55]
Delay discounting (AuC)^†^	− 0.04	0.02	− 1.81	.071	0.96	[− 0.09, 0.01]

Random effects	Estimate	*SD*
Intercept error variance (country)	1.17	1.08

^**†**^The variable was scaled to improve model fit. AuC = Area-under-the-curve; CI = confidence interval; OR = odds ratio; SD = standard deviation; SE = standard error of the mean. Female was used as the reference category for gender. Undergraduate level of education was used as the reference category for level of education. Unvaccinated was used as the reference category for vaccination status

## Discussion

The current research investigated delay discounting as a predictor of PHMs, including protective behaviors with more delayed benefits (i.e., vaccination) and those with more immediate benefits (i.e., cleaning, physical distancing, and mask-wearing). A large multinational sample allowed us to detect differences in PHM compliance across 13 countries. As predicted, discounting of delayed rewards was a significant positive predictor of vaccination status: individuals who tend to choose more delayed rewards over smaller immediate rewards (i.e., far-sighted thinking) were more likely to be vaccinated (see also Halilova et al., 2022). Delay discounting was a significant predictor of the frequency of cleaning and handwashing behaviors. Also as predicted, the direction of the relationship was opposite to that for vaccination: individuals who tend to choose more immediate rewards over larger later rewards (i.e., near-sighted thinking) engaged in cleaning behaviors more frequently. Near-sighted thinking was also a significant positive predictor of physical distancing in the countries tested, other than the UK, where delay discounting was instead a negative predictor of physical distancing. Interestingly, this was the only country in our sample where there were no physical distancing mandates in place during the time of data collection (Mathieu et al., 2020). Lastly, delay discounting did not significantly predict likelihood of mask-wearing over and above the control variables.

The association between delay discounting and COVID-19 vaccination status extends recent findings in a subsample of the participants tested in the current study (Halilova et al., 2022) and is consistent with findings from other recent studies on the topic (Hudson et al., 2022; Strickland et al., 2022). The results are also consistent with research showing that even though nonmonetary outcomes (e.g., health) are generally discounted more steeply than monetary outcomes (Baker et al., 2003; Odum et al., 2020), discounting has trait-like qualities and may be context-independent (Odum et al., 2020). The findings suggest that delay discounting is an important behavioral economic predictor of compliance with PHMs designed to curb the spread of the virus.

The findings were somewhat more mixed when it comes to other PHMs. As hypothesized, the tendency to choose more immediate smaller rewards over larger later rewards predicted more frequent cleaning and handwashing behaviors across most countries. The findings are consistent with previous research showing a positive association between near-sighted thinking and hand hygiene in Belgium, France, Italy, the Netherlands, Portugal, and Sweden (Calluso et al., 2021; Wismans et al., 2021). Exploration of this relationship across the 13 countries revealed that delay discounting was not a significant predictor of cleaning behaviors in Germany, New Zealand, and Poland. This result could be explained by country-specific variability in compliance with hand hygiene during COVID-19 (Szczuka et al., 2021). Although there is no previous research systematically investigating the relationship between delay discounting and PHMs in Germany and New Zealand, our findings are consistent with previous research by Krawiec et al. (2022) showing no significant relationship between delay discounting and hand hygiene in Poland. This finding helps to rule out study-specific explanations (e.g., differences in research design, language, measures used) for the discrepancies in previous findings (e.g., Calluso et al., 2021; Krawiec et al., 2022; Wismans et al., 2021) related to the relationship between delay discounting and cleaning behaviors. This suggests that there may be a country-level variable moderating this relationship, which can be further explored in future research. For example, it is possible that severity of penalties for mandate violations could be one such variable. To the best of our knowledge, there is no global data source detailing such penalties and the enforcement of penalties varied across jurisdictions and institutions (e.g., workplaces). Future research would benefit from investigating the role of mandate violation penalties and their enforcement on PHMs and its relationship with delay discounting at a more regional level.

We also found that the tendency to choose immediate smaller rewards over larger later rewards predicted more frequent engagement in physical distancing behaviors during COVID-19, but only after the UK was excluded from the analysis due to the absence of physical distancing mandates. The findings are consistent with previous research showing a positive association between near-sighted thinking and willingness to engage in immediate protective measures (Calluso et al., 2021; Wismans et al., 2021). Although unexpected, the finding of a negative relationship between near-sighted decision making and physical distancing in the UK, where there were no physical distancing restrictions in place at the time of data collection in 2021, is also consistent with previous findings in a UK sample by Lloyd et al. (2021). It may be that when free to exercise personal choice over whether to physically distance or not, different factors are considered than when deciding to comply with PHMs that are mandated. Moreover, the removal of mandates may signal that immediate safety concerns have resolved and, thus, perhaps it is not surprising that people who tended to engage in physical distancing in this context were the ones who discounted the future less (i.e., those who tended to prioritize longer-term rewards). It should be noted that this potential moderating effect of mandates on the association between delay discounting and physical distancing limits generalizability of our findings to times when mandates are not in place. Future research would benefit from investigating more directly the moderating effects of various government mandates on the relationship between delay discounting and PHM compliance.

Interestingly, the finding of the positive relationship between delay discounting and physical distancing contradicts the results reported by Agrawal et al. (2023), who observed the opposite effect. One potential explanation for this discrepancy could be the timing of data collection. Agrawal et al. collected their data in 2020, a period before vaccines became widely available, and thus it is likely that at this time, adherence to physical distancing measures and mask-wearing were the primary protective strategies against infection. In contrast, our dataset was collected after vaccines became available, providing participants with more options in terms of engaging in protective behaviors. It is also possible that differences in measures, such as varying monetary amounts on delay discounting tasks and different scales to quantify PHMs, may also have contributed to the discrepant findings. Further exploration and reconciliation of these findings could provide valuable insights into the interplay between temporal discounting and health behaviors in varying contexts.

Although unexpected, the nonsignificant relationship between mask-wearing and delay discounting is consistent with some previous findings (Agrawal et al., 2023; Krawiec et al., 2022), but not others (Byrne et al., 2021; Calluso et al., 2021). One explanation is that compared to others protective measures (e.g., physical distancing and handwashing), mask-wearing mandates are socially enforced as non-compliance is easily detectable, thereby restricting people’s freedom of choice relating to compliance with this PHM. For example, if one is not wearing a mask in a hospital where masks are required, one may be asked to either put on their mask or be denied service. On the other hand, monitoring and enforcing compliance with physical distancing in public venues may be a greater challenge. Compliance with physical distancing, cleaning, and handwashing may therefore depend to a greater extent on one’s decision-making process than compliance with mask-wearing. It is also possible that because mask-wearing behavior is more observable compared to other PHMs, it is more likely to be subject to social conformity (Mladenović et al., 2023). In other words, people’s decisions to wear masks in public places may be influenced to a greater degree by social norms, rather than short- and long-term personal costs and benefits.

Another possible explanation for the different patterns of delay discounting in relation to vaccination vs. cleaning and, when mandates exist, physical distancing is risk compensation, the idea that individuals adjust their behavior to maintain a preferred level of risk (Mantzari et al., 2020). For example, if individuals perceive themselves as being at lower risk of contracting COVID-19 due to being vaccinated, they may engage in riskier behaviors, such as reduced compliance with physical distancing, mask-wearing, or hygiene protocols. We view this explanation as unlikely due to the positive relationship between vaccination and other PHMs. Consistent with other research (e.g., Goldszmidt et al., 2021), we do not observe evidence of risk compensations among participants in our sample.

Patterns of delay discounting might also differ across various PHMs due to the cost of engaging in protective behaviors in the present moment (Petherick et al., 2021). Given that mask-wearing is a relatively low-cost protective behavior, individuals may readily implement it without needing to assess the short- and long-term costs and benefits of that behavior. On the other hand, cleaning and handwashing behaviors may be more time-consuming (i.e., costly) to engage in and require an assessment of costs and benefits (e.g., spending the time engaging in other more enjoyable activities). Mandated physical distancing may be viewed as a higher-cost behavior that requires one to assess costs and benefits of avoiding interacting with others (e.g., missing an important event, working remotely). Additionally, it is worth noting that various PHMs can have short- and long-term gains and losses that may vary at an individual level, and that future research is needed to sort out these complexities.

The mixed findings in relation to different PHMs are consistent with previous research that indicates the importance of distinguishing between different protective behaviors, as some of them may be differently affected by one’s personality, demographic factors (e.g., Choi et al., 2022; MacIntyre et al., 2021), and economic considerations (Petherick et al., 2021). In a recent meta-analysis, intention–behavior relationships of physical distancing, hand hygiene, and mask-wearing were assessed (Liang et al., 2022). Although several studies reported a positive intention–behavior relationship when it comes to physical distancing and hand hygiene, there was a nonsignificant intention–behavior relationship found for mask-wearing (Liang et al., 2022). Overall, the literature suggests that decisions to comply with different PHMs may rely on different mechanisms and be influenced by different factors.

The multinational nature of the sample also allowed us to examine consistency of the relationship between delay discounting and frequency of engaging in PHMs across countries, helping to begin reconciling discrepancies in previous studies and exploring other factors influencing compliance with protective behaviors (e.g., government mandates). For example, examining the relationship across all of the PHMs in the current study, it appears that in Poland there is almost no association between discounting of delayed rewards and PHM compliance, which is consistent with previous literature (Krawiec et al., 2022). This discrepant finding suggests the need for further investigation of the impact of economic and cultural factors that have been previously found to influence delay discounting on PHM compliance (Du et al.., 2002; Ishii et al., 2016; Ruggeri et al., 2022).

## Conclusion and future directions

Overall, delay discounting is a behavioral economic measure that predicts compliance with most COVID-related PHMs, including vaccination, cleaning and handwashing, and, when government restrictions are in place, physical distancing. Temporal delay of protective benefits of PHMs may determine the direction of the relationship between delay discounting and decisions to engage in the PHMs. This is the first study, to our knowledge, demonstrating the different relationships between delay discounting and vaccination acceptance and adherence to other protective measures within the same sample of participants. Delay discounting may be a useful behavioral feature to consider in constructing future forecasting models of the spread of COVID-19, as the existing models have been criticized for lack of integration of behavioral measures accounting for compliance with protective measures against the disease spread (Chen et al., 2020).

Given that delay discounting is a modifiable characteristic, future research should focus on investigating the effectiveness of different interventions targeting delay discounting (e.g., cuing individuals to imagine the future; Bromberg et al., 2017; Ciaramelli et al., 2021; Mok et al., 2020; Rung & Madden, 2018) to improve the compliance with various PHMs. The findings inform future policies designed to encourage PHM compliance and reinforce the utility of behavioral economic measures in orienting people toward making healthier choices that have global societal benefits. Future research would also benefit from empirically testing nonmonetary delay discounting when it comes to vaccination to assess whether individuals place greater emphasis on short-term vs. long-term rewards.

### Supplementary Information

Supplementary Material 1.

## Data Availability

Anonymized, raw, and cleaned data have been deposited in a public repository hosted by the Open Science Framework https://osf.io/nyt4g/?view_only=7482638ff0bd4963867ed3b1d6f0833d. The materials used in this study are either described in the method section or publicly available.

## Abbreviations

PHM: Public health measure
AuC: Area-under-the-curve
IUS-12: Intolerance of Uncertainty Scale-12

## Footnotes

1 A subset of the data (n = 5,193) collected from June 27, 2021 to July 16, 2021 was previously reported in Halilova et al., 2022.

2 USA, Canada, Mexico, UK, Italy, France, Portugal, the Netherlands, Spain, Germany, Poland, Australia, New Zealand, and South Africa.
