# Supplementary material for: Assessing the relationship between delay discounting and decisions to engage in various protective behaviors during COVID-19
Source: Cogn Res Princ Implic. 2024 Jun 18;9:38. doi: 10.1186/s41235-024-00566-6 (PMC11183030; doi:10.1186/s41235-024-00566-6)
Supplement: Supplementary file 1 — Supplementary Material 1. [file 41235_2024_566_MOESM1_ESM.docx]

**Supplementary Materials**

**Table S1.** Descriptive Summary by Country

|  | **Australia**  **n = 690** | **Canada**  **n = 1025** | **France**  **n = 97** | **Germany**  **n = 234** | **Italy**  **n = 396** | **Mexico**  **n = 451** | **Netherlands**  **n = 134** |
| --- | --- | --- | --- | --- | --- | --- | --- |
| **Age** | 29.26 (11.26) | 29.07 (10.29) | 28.99 (9.45) | 27.45 (9.09) | 28.41 (8.05) | 26.74 (6.70) | 27.35 (9.53) |
| **Gender (Female/Male/Non-binary)** | 450/ 226/ 13 | 667/ 327/ 24 | 42/ 54/ 0 | 139/ 89/ 3 | 172/ 211/ 7 | 209/ 230/ 11 | 59/71/3 |
| **Education Level (no education/primary /secondary /undergraduate /postgraduate)** | 0/ 1/ 280/ 298/ 110 | 1/ 1/ 345/ 562/ 110 | 0/ 0/ 13/ 47/ 36 | 2/ 0/ 103/ 83/ 43 | 1/ 1/ 162/ 160/ 66 | 2/ 0/ 82/ 318/ 48 | 0/ 0/ 36/ 62/ 35 |
| **Income (0-100)** | 35.86 (24.49) | 37.14 (23.70) | 32.84 (27.20) | 32.24 (24.21) | 33.53 (21.59) | 43.56 (23.01) | 39.30 (25.46) |
| **Essential Workers *(% yes)*** | 26% | 32% | 14% | 14% | 10% | 17% | 19% |
| **Intolerance of Uncertainty** | 34.48 (9.29) | 35.11 (9.27) | 34.68 (9.61) | 35.53 (8.92) | 36.54 (8.60) | 34.62 (8.90) | 33.16 (8.59) |
| **Area-under-Curve** | 0.39 (0.25) | 0.42 (0.25) | 0.40 (0.24) | 0.44 (0.25) | 0.41 (0.26) | 0.30 (0.24) | 0.48 (0.25) |
| **Psychological Distress** | -0.03 (1.92) | 0.08 (1.90) | -0.03 (1.96) | 0.12 (1.83) | 0.15 (1.88) | 0.07 (1.86) | -0.35 (1.65) |
| **Cleaning *(Median)*** | 4 | 4 | 4 | 4 | 5 | 5 | 4 |
| **Physical Distancing *(Median)*** | 5 | 5 | 5 | 4 | 4 | 5 | 5 |
| **Mask-Wearing *(Median)*** | 3 | 3 | 3 | 3 | 3 | 4 | 2 |
| **Vaccination Status *(% yes)*** | 24% | 89% | 52% | 79% | 61% | 33% | 65% |

|  | **New Zealand**  **n = 191** | **Poland**  **n = 639** | **Portugal**  **n = 403** | **Spain**  **n = 445** | **United Kingdom**  **n = 987** | **United States**  **n = 1234** |
| --- | --- | --- | --- | --- | --- | --- |
| **Age** | 30.53 (11.49) | 23.44 (5.76) | 25.61 (7.04) | 30.25 (10.54) | 31.38 (11.67) | 29.56 (11.13) |
| **Gender (Female/Male/Non-binary)** | 125/ 59/ 2 | 220/ 392/ 14 | 185/ 210/ 2 | 201/229/11 | 679/ 284/ 10 | 813/376/33 |
| **Education Level (no education/primary /secondary /undergraduate /postgraduate)** | 0/ 1/ 54/ 98/ 34 | 1/ 5/ 316/ 212/ 93 | 0/ 0/ 119/ 190/ 89 | 0/ 1/ 115/ 227/ 98 | 1/ 2/ 373/ 442/ 155 | 1/ 5/ 418/ 616/ 185 |
| **Income (0-100)** | 38.99 (24.11) | 36.88 (24.14) | 35.86 (24.64) | 36.92 (23.43) | 37.27 (23.80) | 36.03 (24.44) |
| **Essential Workers *(% yes)*** | 20% | 9% | 18% | 9% | 33% | 28% |
| **Intolerance of Uncertainty** | 32.80 (9.30) | 36.15 (8.42) | 36.32 (9.12) | 34.38 (9.19) | 34.87 (9.72) | 35.38 (9.78) |
| **Area-under-Curve** | 0.38 (0.22) | 0.36 (0.25) | 0.41 (0.27) | 0.40 (0.24) | 0.39 (0.23) | 0.40 (0.25) |
| **Psychological Distress** | -0.46 (1.58) | 0.29 (1.78) | 0.08 (1.91) | -0.17 (1.84) | -0.08 (1.91) | -0.09 (1.94) |
| **Cleaning *(Median)*** | 4 | 4 | 5 | 4 | 5 | 4 |
| **Physical Distancing *(Median)*** | 3 | 4 | 4 | 4 | 5 | 4 |
| **Mask-Wearing *(Median)*** | 0 | 3 | 4 | 4 | 3 | 3 |
| **Vaccination Status *(% yes)*** | 19% | 70% | 14% | 43% | 86% | 79% |

**Table S2.** COVID-19 mandates by country derived from Our World in Data (Mathieu et al., 2020).

| **Country** | **Vaccination** | **Stay-at-home requirements** | **Cancellation of Public Events** | **Mask-Wearing** |
| --- | --- | --- | --- | --- |
| **Australia** | Vulnerable + some other (June 27 – June 29, 2021); Universal (June 30 – August 31, 2021) | Required (except essentials) | Required | Required in all public spaces |
| **Canada** | Universal | Recommended | Required June 27 – July 15, 2021; Recommended July 16 – August 25, 2021; Required August 26 – 31, 2021 | Required in all public spaces |
| **France** | Universal | No measures | Required | Required in all public spaces |
| **Germany** | Universal | Required (except essentials) | Required | Required in some public spaces |
| **Italy** | Universal | Required (except essentials) | Required | Required in some public spaces |
| **Mexico** | All Vulnerable groups (June 27 – June 30, 2021); Universal (July 1 – August 31, 2021) | Recommended June 27 – July 25, 2021 and required (except essentials) July 26 - August 31, 2021 | June 27 – August 25, 2021 Recommended; Required August 26 – 31, 2021 | Required outside-the-home at all times |
| **The Netherlands** | Universal | Recommended | Recommended | Required in some public spaces |
| **New Zealand** | All vulnerable groups (June 27 – July 27, 2021); Vulnerable + some other (July 27 – August 31, 2021) | No measures until August 16, 2021; required (except essentials) August 17 - 31, 2021 | No measures until August 16, 2021; Required between August 17 - 31, 2021 | Required in all public spaces |
| **Poland** | Universal | Recommended | Recommended | Required in some public spaces |
| **Portugal** | Vulnerable + some other (June 27 – July 27, 2021); Universal (July 28 – August 31, 2021) | Recommended June 27-July 1,2021 and required (except essentials) July 2 - August 31, 2021 | Required | Required in all public spaces |
| **Spain** | All vulnerable groups June 27 – July 10, 2021; Vulnerable + some other July 11 – July 24, 2021; Universal July 24 – August 31, 2021 | Recommended June 27-July 15, 2021 and required (except essentials) July 16 - August 31, 2021 | Required | Required in some public spaces |
| **United Kingdom** | Universal | No measures | Recommended | Required in some public spaces |
| **United States** | Universal | Recommended | Required June 27 – July 7, 2021; Recommended July 8 – August 23, 2021; Required August 24 – 31, 2021 | Required in some public spaces |

**Figure S1**. Average COVID-19 Stringency Index between June 27 – August 31, 2021 by country derived from Our World in Data (Mathieu et al., 2020).


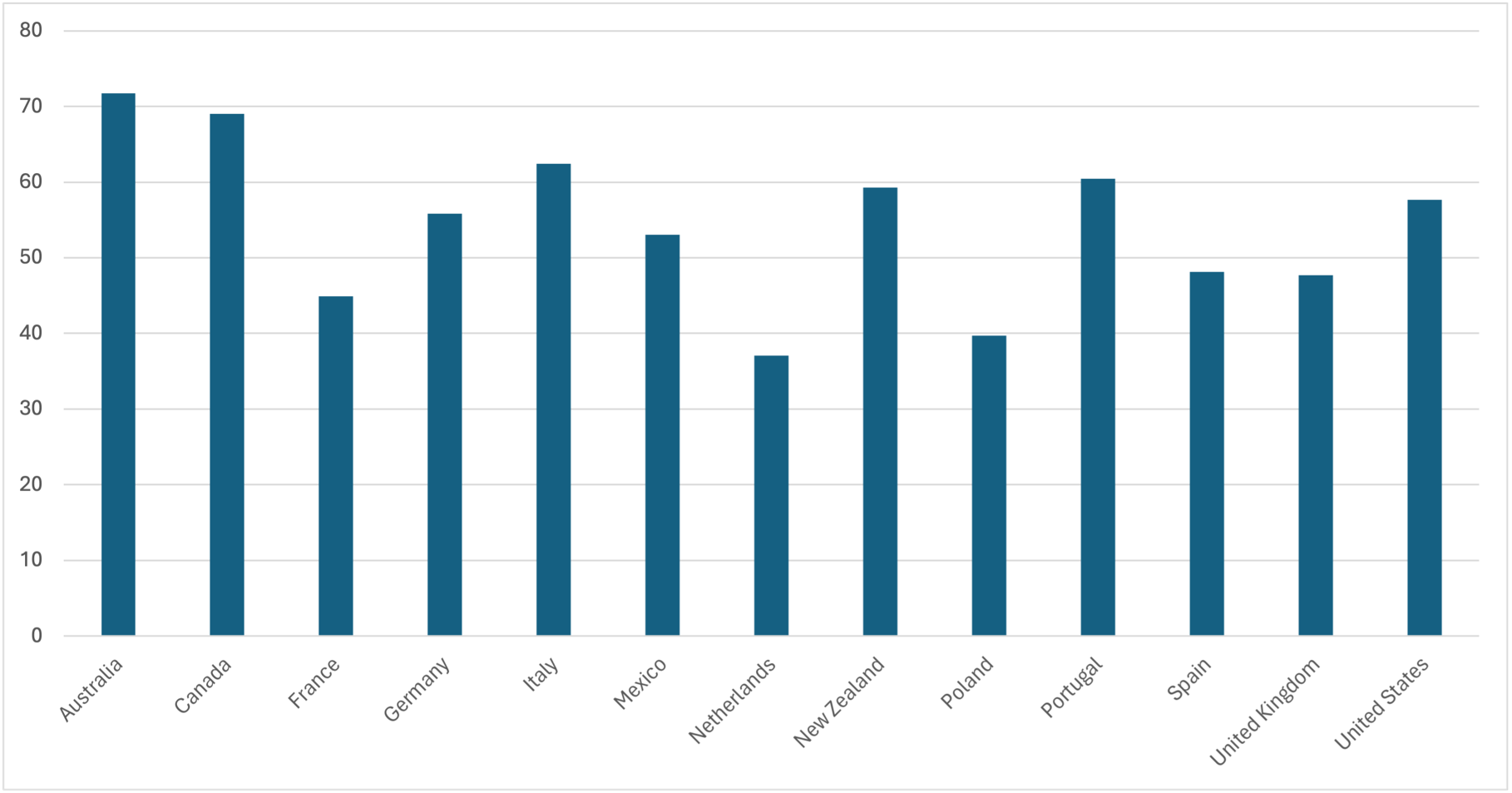


*Note.* The nine metrics used to calculate the **Stringency Index** are: school closures; workplace closures; cancellation of public events; restrictions on public gatherings; closures of public transport; stay-at-home requirements; public information campaigns; restrictions on internal movements; and international travel controls (100 = strictest response)
